# Using qualitative interviews with nihr crn research midwives to refine trial recruitment methods

**DOI:** 10.1186/1745-6215-16-S2-P74

**Published:** 2015-11-16

**Authors:** Katharine Foster, Katharine Bowker, Sophie Orton, Felix Naughton, Sue Cooper, Tim Coleman

**Affiliations:** 1 University of Nottingham, Nottingham, UK; 2 University of Cambridge, Cambridge, UK

## Background

Smoking during pregnancy increases the risks of many pregnancy-related complications. MiQuit is a tailored, self-help, text-message intervention developed to help pregnant smokers to stop. A pilot RCT investigated feasibility of recruitment of women attending hospital antenatal clinics by using NIHR Clinical Research Network (CRN) research midwives (RMs); findings will be used to plan a future definitive trial to investigate MiQuit efficacy. We aimed to describe the facilitators and barriers to trial recruitment that RMs perceived, and relate these to key recruitment processes.

## Methods

We conducted 14 semi-structured telephone interviews with RMs from 13 of 15 pilot trial recruiting centres. All interviewees had been involved in local study set-up, participant recruitment and follow-up. Data were transcribed verbatim, transcripts were coded simultaneously and inductive thematic analysis was used to analyse data.

## Results

Emergent findings suggest a number of pertinent themes, most notably with participant identification and screening. For example, experienced RMs generally felt that a screening questionnaire intended to be given to all women attending antenatal clinics, and therefore prevent smokers from feeling unfairly targeted, was ineffective when used in the proposed manner and could cause greater discomfort on both sides. Instead, RMs indicated that they preferred to pre-identify smokers and discreetly approach them.

## Conclusions

Qualitative exploration of research staff views can help maximise the value of pilot work, identifying key aspects of trial design and conduct that may affect recruitment. We will discuss how these findings can assist planning a definitive trial, highlighting issues which may be generalisable to other studies.

